# A low voltage activated Ca^2+^ current found in a subset of human ventricular myocytes

**DOI:** 10.1080/19336950.2020.1794420

**Published:** 2020-07-20

**Authors:** Xin Zhang, Yijia Li, Xiaoying Zhang, Valentino Piacentino, David M. Harris, Remus Berretta, Kenneth B. Margulies, Steven R. Houser, Xiongwen Chen

**Affiliations:** a Department of Infection Diseases The First Affiliated Hospital of China Medical University, Shenyang China; b Department of Physiology and Cardiovascular Research Center, Temple University Lewis Katz School of Medicine, Philadelphia, PA, USA; c Department Grand Strand Surgical Care, Grand Strand Regional Medical Center, Myrtle Beach, SC; d College of Medicine, University of Central Florida, Orlando, Florida, USA; e Department of Medicine, University of Pennsylvania, Philadelphia, PA, USA

**Keywords:** Heart failure, low voltage activated Ca^2+^ current, Cav1.3, T-type calcium channel, ventricular myocytes, left ventricular assist device

## Abstract

Low voltage activated (I_Ca-LVA_) calcium currents including Cav1.3 and T-type calcium current (I_Ca-T_) have not been reported in adult human left ventricular myocytes (HLVMs). We tried to examine their existence and possible correlation with etiology and patient characteristics in a big number of human LVMs isolated from explanted terminally failing (F) hearts, failing hearts with left ventricular assist device (F-LVAD) and nonfailing (NF) human hearts. LVA (I_Ca-LVA_) was determined by subtracting L-type Ca^2+^ current (I_Ca-L_) recorded with the holding potential of −50 mV from total Ca^2+^ current recorded with the holding potential of −90 mV or −70 mV. I_Ca- LVA_ was further tested with its sensitivity to 100 µM CdCl_2_ and tetrodotoxin. Three HLVMs (3 of 137 FHLVMs) from 2 (N = 30 hearts) failing human hearts, of which one was idiopathic and the other was due to primary pulmonary hypertension, were found with I_Ca-LVA_. I_Ca-LVA_ in one FHLVM was not sensitive to 100 µM CdCl_2_ while I_Ca-LVA_ in another two FHLVMs was not sensitive to tetrodotoxin. It peaked at the voltage of −40~-20 mV and had a time-dependent decay faster than I_Ca-L_ but slower than sodium current (I_Na_). I_Ca-LVA_ was not found in any HLVMs from NF (75 HLVMs from 17 hearts) or F-LVAD hearts (82 HLVMs from 18 hearts) but a statistically significant correlation could not be established. In conclusion, I_Ca-LVA_ was detected in some HLVMs of a small portion of human hearts that happened to be nonischemic failing hearts.

## Introduction

Calcium (Ca^2+^) influx through calcium channels plays important roles in the heart. It not only mediates excitation-contraction coupling, but also regulates many other processes such as transcription, myocyte death and hypertrophy [1]. Two types of calcium channels, high-voltage activated (mainly Cav1.2 L-type calcium channel, LTCC) and low voltage activated calcium channels (I_Ca-LVA_, Cav1.3 L-type and Cav3 T-type calcium channels, TTCCs), are present at the gene expression and functional levels in cardiomyocytes [2,3]. Three types of TTCCs, Cav3.1 (a1 G), Cav3.2 (a1 H) and Cav3.3 (a1I), are encoded by three genes, CACNA1 G, CACNA1 H, and CACNA1I, respectively. The former two are the ones expressed in the heart [2,3]. As with Cav3.1 and Cav3.2, Cav1.3 is normally present in pacemaker tissues participating cardiac rhythm generation and atria but has not been reported in mammalian ventricular myocytes [4].

It has been reported that the expression of TTCCs in cardiomyocytes depends on species, developmental stages, cardiac regions, disease states and cell cycles of cardiomyocytes [2,5]. TTCCs are ubiquitously expressed in cardiomyocytes of all chambers in mammalian fetal hearts and may participate in fetal myocyte excitation-contraction coupling [6]. In fetal mouse hearts, the expression of Cav3.1 and Cav3.2 are temporally and spatially different. For example, in mice, Cav3.1 appears at E8.5 in all myocytes and outflow tract cells while Cav3.2 mRNA expresses at a later stage and has the highest expression in the atrioventricular canal [7]. In human ventricles, Cav3.2 mRNA is present in fetal hearts and decreases during heart development [8]. After birth, TTCC expression remains in the automatic tissues and the conduction system but disappears in other regions of the heart like the ventricles. As TTCCs remain in the sinoatrial and atrioventricular node and the conduction system, it has been proposed that the major function of TTCCs in adult hearts is to initiate cardiac rhythm and regulate cardiac conduction [2]. Recently, we showed that TTCCs play an important role in heart rate regulation by the sympathetic/β-adrenergic system [9]. I_Ca-T_ expression in rat atrial myocytes decreases during postnatal development [10,11]. In general TTCCs are absent in adult ventricular myocytes (VMs) of mammals with the exception of a few species including guinea pigs [12,13]. However, thus far, there has been no I_Ca-T_ reported in adult human ventricular myocytes [14].

While TTCCs participates in excitation-contraction coupling in fetal ventricular myocytes [6], their role in adult ventricular myocytes is still elusive. I_Ca-T_ in adult guinea pig ventricular myocytes participates in excitation-contraction coupling but the efficiency is low [15]. With cardiomyocyte specific transgenic expression of Cav3.1 in mice, we showed that T-type Ca^2+^ current is ineffective to load the SR, neither does it induce Ca^2+^ release from the SR [16]. Interestingly, I_Ca-T_ expression was found to be associated with the S phase of cell cycle in cultured neonatal rat ventricular myocytes [17]. We have reported that TTCCs were expressed in high percentage of mononucleated ventricular myocytes of adolescent feline hearts, which may be related to their potential of proliferation [18]. I_Ca-T_ has been reported to reappear in ventricular myocytes from diseased hearts induced by pressure-overload, post myocardial infarction, and dystrophin deficiency [2,19]. It is not entirely clear the significance of reappearance of TTCCs in stressed hearts. Cav3.2 may provide Ca^2+^ signal for cardiac hypertrophy [20] while Cav3.1 mediated Ca^2+^ influx is antihypertrophic via the NO/cGMP/PKG signaling pathway [21].

Cav1.3 belongs to L-type Ca^2+^ channels and is predominantly expressed in pacemaking tissues and weakly in the atria. Its existence in human ventricles is generally considered absent [4,22] but Lu et al. detected Cav1.3 proteins in human ventricular tissue [23]. Compared to Cav1.2 channels, Cav1.3 channels are activated at quite low voltages overlapping the range of activation voltages for Cav3, inactivate more slowly and less sensitive to dihydropyridine LTCC blockers than Cav1.2 [24]. Yet, Cav1.3 current is sensitive to the blockade by 100 µM CdCl_2,_ as is Cav1.2 current [25].

It has been reported that there is another calcium permeable channel activated at low voltage in sodium-free bath solution, the TTX-sensitive sodium channel, in cardiac myocytes. The current through this type of channel was termed as TTX-sensitive calcium current (I_Ca(TTX)_). I_Ca(TTX)_ was first reported in rat ventricular myocytes [26] and also found in human atrial myocytes [27]. However, its existence has been controversial [28].

This study is to determine if there is low-voltage activated calcium currents (I_Ca-LVA_) in human ventricular myocytes by examining a large number of failing and nonfailing HLVMs and to characterize this low-voltage activated calcium current. We found a small portion of failing human hearts had some LVMs expressing a low-voltage activated, partially or fully inactivated by the holding potential of −50 mV but TTX-insensitive current. This current was not detected in HLVMs from nonfailing hearts or failing hearts with LVAD support.

## Methods

### Myocyte isolation

Human left ventricular myocytes (HLVMs) were isolated from the midwall of the LV free wall of 17 nonfailing (NF), 30 failing (F) and 18 failing human hearts with left ventricular assist device (LVAD) support as described previously [29] and used within 12 hours after isolation. Failing and LVAD–supported failing human hearts were obtained from the Temple Cardiac Transplant Team at the time of cardiac transplantation. Nonfailing hearts were donor hearts unsuitable for transplantation. Our protocol was approved by Temple University Institutional Review Board. Patient characteristics were presented in **Table 1** with detailed information in **Table 3**.

**Table 1.** Patient characteristics.

Patient	Etiology	Male/ Female	Age (y)	CHF Duration (months)	Duration of LVAD (days)	LVEF (%)
NF (*N = 17*)	-	9/8	60.2 ± 3.9	0	-	55.2 ± 1.9
F (*N = 30*)	Idiopathic 7 Ischemic 18 Hypertension 2 Valvular 2 Dilated cardiomyopathy 1	22/8	53.5 ± 2.0	62.1 ± 15.1	-	13.6 ± 2.0***
LVAD (*N = 18*)	Idiopathic 7 Ischemic 8 Hypertension 1 Postpartum 1 Toxic 1	10/8	52.9 ± 2.5	69.7 ± 13.6	194 ± 59.9	14.7 ± 3.9*** (pre-LVAD value)

Data from individual patients, including medications is presented in the on-line supplement. ***: *p < 0.001* versus NF (one-way ANOVA).

**Table 3.** Characteristics of nonfailing donors and failing and failing-LVAD patients.

Patient	Age (y)		Sex		Body Weight (kg)	Heart Weight (g)		LVEF (%)	Medication	
NF Donors (N = 17)										
1	58		f		55	378		60	Db,BB,N	
2	16		f		52	373		52.5	Db,M	
3	66		m		70	518		60	N	
4	79		m		77	583		-	ACEI,Du	
5	64		f		48	303		55	-	
6	65		m		88	534		57.5	-	
7	62		f		64	369		45	Dg	
8	40		m		85	-		50	BB	
9	72		m		77	495		-	ACEI,BB,CaB	
10	68		m		66	471		-	BB,AB	
11	59		m		113	530		55	BB,AB	
12	51		f		81	378		62.5	ACEI,BB,AB	
13	76		m		70	365		-	-	
14	43		f		70	335		-	-	
15	61		m		-	472		-	-	
16	72		f		-	374		-	-	
17	72		f		-	411		-	-	
Mean±SEM	60 ± 4				73 ± 4	431 ± 20		55 ± 1		
Patient	Age (y)	Sex	Body Weight (kg)	Heart Weight (g)	CHF Duration (mo)	Etiology	CI (L/min)	LVEF (%)	Medication	
HF patients (N = 30)										
1	52	f	92	762	144	Idio	2.57	22.5	M,ACEI,ANGI,Dg,Du,Am	
2	30	f	59	429	-	Hypertrophic	-	-	M,BB,Dg,Du	
3	62	m	91	617	-	Isch	-	17.5	ACEI,BB,CaB,N,Dg,Du,Am	
4	20	m	64	377	2	Idio	3.6	7.5	Db,ANGI,Dg,Du	
5	62	m	67	417	-	Isch	4.51	17.5	M,ACEI,N,Dg,Du	
6	48	f	66	-	-	Val	2.6	10	M,ACEI,ANGI,N,Du,Am	
7	62	m	86	668	-	Isch	4.6	17.5	M,ANGI,BB,N,Hy,Du	
8	46	m	77	451	77	Idio	3.1	5	M,ACEI,ANGI,CaB,Dg,Du	
9	45	m	74	488	-	Isch	1.4	10	Db,M,ACEI,ANGI,N,Dg,Du,Am	
10	65	m	79	604	57	Isch	3.05	7.5	Db,M,ANGI,Dg,Am	
11	59	f	68	527	-	Isch	2.2	5	Db,M,ACEI,N,Dg	
12	64	m	78	630	20	Isch	3	12.5	Db,M,ACEI,N,Dg	
13	62	m	80	599	43	Isch	3	12.5	Db,M,ANGI,CaB,N,Hy,Dg,Du	
14	37	m	93	575	-	Idio	1.5	12.5	Db,M,ACEI,CaB,N,Hy,Du,Am	
15	58	m	53	493	-	Isch	-	12.5	Db,M,ACEI,Am	
16	61	m	76	470	-	Isch	2.1	10	Db,M,ACEI,ANGI,N,Dg,Du,Am	
17	-	-	-	-	-	Htn	-	-	-	
18	57	m	69	587	-	Isch	2	7.5	M,ACEI,ANGI,BB,N,Dg,Du	
19	52	m	111	553	-	Isch	2.12	12.5	M,ACEI,N,Dg,Du,Am	
20	62	f	90	839	132	Isch	3.1	12.5	Db,Du	
21	61	f	76	374	-	Isch	4.75	57.5	BB,CaB,N,Du	
22	50	m	73	366	36	Idio	-	7.5	ACEI,BB,Dg,Du	
23	57	m	-	611	-	Idio	-	7.5	-	
24	47	f	59	337	72	DCM	1.9	15	Db,M,ACEI,BB,Du	
25	56	m	72	1034	4	Isch	-	7.5	Db,M,ACEI,Dg,Du,Am	
26	58	m	85	761	-	Valv	2.28	7.5	M,N,Hy,Dg,Du,Am	
27	41	m	-	550	-	PPHTn	1.7	30	Db,Am	
28	64	m	77	492	96	Isch	-	10	Db,M,ACEI,N,Dg,Du	
29	59	m	100	-	-	Isch	-	12.5	ACEI,BB,N,Du,Am	
30	55	m	-	602	-	Isch	-	12.5	ACEI,BB,N,Hy,Du	
Mean±SEM	54 ± 2		78 ± 2	563 ± 29	62 ± 9		3 ± 0	14 ± 2		
Patient	Duration of LVAD (days)	Age (y)	Sex	Body Weight (kg)	Heart Weight (g)	CHF Duration (month)	Etiology	Pre-LVAD CI (L/min)	LVEF (%)	Medication
LVAD patients (N = 18)										
1	-	60	m	79	26	136	Isch	1.8	10	-
2	28	48	m	68	22	172	Isch	2.3	7.5	-
3	83	59	m	74	24	60	Idio	1.8	7.5	ANGI,Am
4	540	66	f	72	31	-	Isch	1.9	35	Db,BB,AB,CaB,N
5	130	54	m	80	24	105	Idio	3.2	7.5	-
6	160	56	f	63	26	57	Isch	1.5	7.5	ANGI,AB,N,Am
7	8	41	m	93	27	3	Isch	-	15	M,BB,N
8	23	54	m	79	24	7	Idio	2	-	N,Dg,Du,Am
9	-	34	f	66	27	108	PP	-	7.5	ACEI,ANGI,BB,Dg,Du
10	164	53	f	65	26	-	Htn	1	7.5	M,Du
11	6	39	m	102	37	58	Idio	1.7	10	-
12	-	63	m	76	25	54	Isch	2.58	12.5	M,ACEI,Dg
13	390	31	f	70	24	1	Idio	2.24	42.5	Dg
14	4	56	m	89	33	94	Isch	3.2	12.5	M,Dg
15	473	53	f	73	26	87	Idio	-	5	CaB
16	508	57	m	81	28	94	Isch	2.2	7.5	BB,AB,Du
17	-	66	f	75	25	-	Idio	-	10	Dg,Du
18	-	63	f	-	-	9	Toxic	-	7.5	CaB,Am
Mean ±SEM	194 ± 49	53 ± 2		77 ± 2	27 ± 1	70 ± 12		2 ± 0	15 ± 3	

AB, α-adrenergic blocker; ACEII, angiotensin-converting enzyme inhibitor; Am, amiodarone; ANGI, angiotensin II inhibitor (angiotensin receptor blocker); BB, β-adrenergic blocker; CaB, calcium channel blocker; CHF, congestive heart failure; CI, cardiac index; Db, dobutamine; DCM, dilated cardiomyopathy; Dg, digoxin; Dop, Dopamine; Du, diuretic; Htn, hypertension; Hy, Hydralazine; Idio, Idiopathic cardiomyopathy; Isch, ischemic cardiomyopathy; LVEF, LV ejection fraction; M, Milrinone; N, nitrate; PPHTn, Primary pulmonary hypertension; PP, postpartum; -, unknown; Valv, valvular cardiomyopathy.

### Cav3.2 transgenic mouse models and ventricular myocyte isolation

To compare I_Ca-T_ expressed in native cardiomyocytes, a mouse model with cardiac-specific (controlled by α-MHC promoter) and conditional (tet-off, controlled by doxycycline, DOX) overexpression of human Cav3.2 (Genebank ID: NM_021098) was established with the bitransgenic system developed by Sanbe A et al. [30] and gifted by the Molkentin group at University of Cincinnati. Ventricular myocytes (VMs) was isolated as described previously [31]. All animal work was approved by Institutional Animal Use and Care Committee of Temple University.

### Ca^2^^±^ current measurements

Whole cell Ca^2+^ currents (I_Ca-L_) were measured in Na^+^- and K^+^-free solutions at 37°C using techniques described in detail previously [9,16,18,21,32]. Briefly, myocytes were added into a water-heated chamber mounted on an inverted microscope (Nikon, Japan) and were initially perfused with a physiological salt solution containing (in mmol/L): NaCl 150, KCl 5.4, CaCl_2_ 1, MgCl_2_ 1.2, glucose 10, sodium pyruvate 2, and HEPES 5 (pH7.4) at 37°C. Low resistance (1–3 MΩ) micropipettes filled with a solution containing (in mmol/L) Cs-aspartate 130, NMDG 10, TEA-Cl 20, Tris-ATP 2.5, Tris-GTP 0.05, MgCl_2_ 1, EGTA 10, pH 7.2, were used for whole cell voltage clamp experiments. The overall junction potential was around −10 mV and not corrected in the analysis. Once a gigaohm seal was obtained, the membrane was ruptured for dialysis while the voltage was set at −70 mV. After myocytes were dialyzed with the internal solution and perfused with the physiological salt solution for 10 minutes, the bath solution was switched to a Na^+^ and K^+^ free bath solution containing (in mmol/L): NMDG 150, CaCl_2_ 2, CsCl 5.4, MgCl_2_ 1.2, Glucose 10, HEPES 5, 4-AP (4-aminopyridine) 2 (pH = 7.4). Three minutes of high-flow (5 mL/min) washing with the Na^+^ and K^+^ free bath solution was done to completely switch out Na^+^ containing physiological solution. Discontinuous switching voltage clamp was achieved with an Axoclamp-2B (Axon Instruments) amplifier. Vm and Im output bandwidth was set at 10 KHz. The switching rate was set at least 7 kHz. The gain was set at least 3 (normally 5–12) to quickly charge and discharge the membrane and to minimize the effect of capacitance charging/discharging currents on recorded currents (mostly achieving 90% voltage control within 2 ms). The Clampex 8 software (Axon Instrument) was used to control the amplifier and to acquire data with a Digidata 1200 (Axon Instruments) analog-to-digital converter. To define the total I_Ca_–voltage relationship, the membrane potential was held at – 70 mV or −90 mV and then depolarized in a 10 mV-increment. The existence of I_Ca-LVA_ was examined retro-respectively and the correlation of its existence to cell and patient characteristics were tested. In one myocyte with I_Ca-LVA_ identified, I_Ca-_voltage relationships were examined with holding potentials at both −90 mV and −50 mV in a 10-mV increment in the presence of 50 μM TTX or 100 µM Cd^2+^. Currents were recorded and analyzed offline with Clampfit 8. A subtraction of the average of the tail current of last two milliseconds of the depolarization from the peak was done before the construction of I_Ca_-voltage relationships. The decay of I_Ca-L_ and I_Ca-LVA_ currents was fitted with a double- or single-exponential decay function built in Clampfit 8, respectively. Minimal sum of squared errors and maximal correlation coefficient (>0.90) were used to determine the goodness of the fitting. Time constants (fast time constants (τ_f_) and slow time constants (τ_s_) for the double-exponential decay function, a single time constant (τ) for the single-exponential decay function) were obtained from the fitting.

### I_Na_ recording and analysis

To simulate potential residual Na^+^ current and contaminating I_Na_, we added 5 mM Na^+^ in the bath solution used for I_Ca_ recording (composition in mM: NaCl 5, NMDG 150, CaCl_2_ 2, CsCl 5.4, MgCl_2_ 1.2, Glucose 10, HEPES 5, 4-AP 2 (pH = 7.4). In a HLVM without I_Ca-LVA_, I_Na_ was recorded with I_Ca-L_ when the myocyte was held at the V_h_ of −90 mV and depolarized with 400 ms test pulses in a 10 mV increment while I_Ca-L_ was recorded with I_Na_ inactivated by the V_h_ = −50 mV. I_Na_ was obtained by subtracting I_Ca-L_ recorded at V_h_ = −50 mV from I_Ca-L_+ I_Na_ recorded at V_h_ = −90 mV. The current-voltage (I–V) relationship of I_Na_ was constructed by plotting peak I_Na_ versus test potentials. The inactivation of I_Na_ was fit with a single exponential decay function built in Clampfit 8 from the peak to 200 ms thereafter. Minimal sum of squared errors and maximal correlation coefficient (>0.90) were used to determine the goodness of the fitting.

### Statistics

Data in the text and tables are reported as mean±SEM. One-way ANOVA was used to detect difference between patient parameters of the nonfailing, failing, and LVAD-failing groups with SAS 9.0 (SAS Institute Inc., Cary, NC). A *p* value of 0.05 was considered significant. Contingency table was used to determine if the occurrence of I_Ca-LVA_ in failing vs. nonfailing human hearts or ventricular myocytes was significantly different.

## Results

### I_Ca-LVA_ existence and its association with etiologies

We totally examined 75 nonfailing LVMs from 17 NF hearts, 137 LVMs from 30 F hearts and 82 LVMs from 18 LVAD-support failing hearts for the existence of I_Ca-LVA_ currents. Of these failing hearts, 7 were idiopathic, 18 ischemic, 2 hypertensive, 2 related to valvular diseases, and 1 with dilated cardiopathy. Of these LVAD-supported failing hearts, 7 were idiopathic, 8 ischemic, 1 hypertensive, 1 with postpartum heart failure, and 1 due to drug toxic. Three LVMs with I_Ca-LVA_ were all found in failing hearts (2 out of 30 hearts, 6.7%) but in none of nonfailing (0 out 18 hearts) and failing with LVAD-support (0 out of 18) hearts (**Table 2**). One LVM with I_Ca-LVA_ was found among 7 examined LVMs (14.3%) from a failing heart with hypertrophic cardiomyopathy (heart #2 in “HF Patients” table of Table 3, female). Two LVMs out of 8 examined LVMs (25%) from a failing heart with primary pulmonary hypertension (heart #27 in “HF Patients” table of Table 3, male) were found with I_Ca-LVA_.

**Table 2.** Percentage of hearts and HLVMs with I_Ca-LVA_ current, in NF, F and LVAD hearts and HLVMs.

	Total Heart Number	Hearts with I_Ca-LVA_	% of hearts with I_Ca-LVA_	Total Cells Examined	Cells with I_Ca-LVA_	Percentage of HLVMs with I_Ca-LVA_
Nonfailing	17	0	0%	75	0	0%
Failing	30	2	6.7%	137	3	2.18%
Failing-LVAD	18	0	0%	82	0	0%

### I_Ca-LVA_ current in a failing HLVM is Cd^2^^±^ insensitive but inactivated by a holding voltage of −50 mV

**Figure 1.**
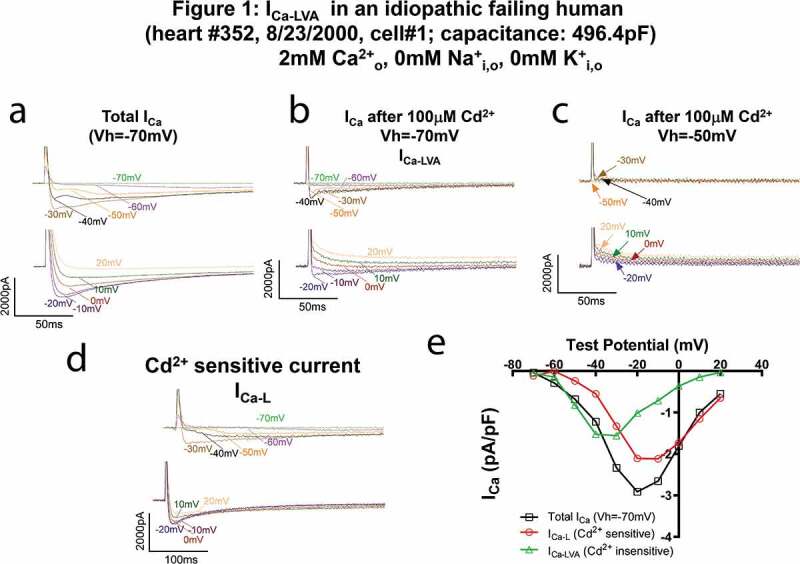
I_Ca-LVA_ in a failing HLVM from a hypertrophic cardiomyopathy heart is insensitive to 100 µM CdCl_2_ but inactivated by the holding potential of −50 mV. (a–d). Current traces at different test voltages (in 10 mV increment from the holding potential, not adjusted for junctional potential) of total I_Ca_ recorded from the holding potential of −70 mV (a), of residual I_Ca_ recorded with the holding potential of −70 mV after the application of 100uM Cd^2+^ (b, presumably I_Ca-T_), of currents recorded with the holding potential of −50 mV after the application of 100uM Cd^2+^ (c), and of Cd^2+^-sensitive currents (differential current obtained by subtracting total I_Ca_ from Cd^2+^ insensitive current, presumably I_Ca-L_, d). (e). The current (i)-Voltage (v) relationships of total I_Ca_, Cd^2+^ sensitive Ca^2+^ current (I_Ca-L_) and Cd^2+^-insensitive but holding potential sensitive current (likely I_Ca-T_).

Figure 1 shows I_Ca_ recorded in a failing human ventricular myocyte. Total I_Ca_ recorded at −50~-30 mV from the holding potential of −70 mV had two peaks/components (Figure 1a), indicating the co-existence of significant low-voltage (I_Ca-LVA_) and high-voltage (L-type) Ca^2+^ currents in one cell. Since L-type Ca^2+^ current (I_Ca-L_) is sensitive but I_Ca-T_ is insensitive to 100 µM Cd^2+^, we tested the sensitivity of these two Ca^2+^ current components to Cd^2+^. We found that there was Cd^2+^-insensitive Ca^2+^ current (Figure 1b) that peaked at −30 mV and inactivated by holding the cell at −50 mV (Figure 1c). The decay of these currents was faster than the Cd^2+^-sensitive current (Figure 1d). In contrast, the Cd^2+^ sensitive current lost the first hump in the current traces (Figure 1d). It had a threshold for activation at −50 mV and peaked at −10 mV (Figure 1e), which was more negative than the usual activation threshold (~-20 mV) and peak (0–10 mV) voltages in normal human and animal ventricular myocytes. This negative activation and peak could be due to the hyperphosphorylation of LTCC in failing myocytes [29] or a combined Cav1.3 and Cav1.2 currents. The peak amplitude of presumed I_Ca-T_ was substantial even it could be underestimated because the holding potential of −70 mV could inactivate a large fraction of I_Ca-T_.

**Figure 2.**
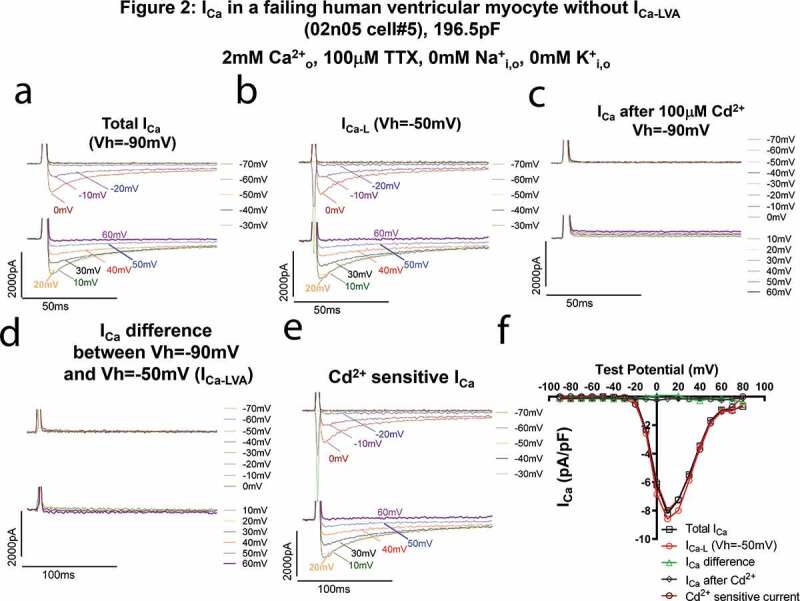
Ca^2+^ currents in a failing HLVM without I_Ca-LVA_. All I_Ca_ was abolished by 100 µM CdCl_2_. (a–d). Current traces at different test voltages (in a 10 mV-increment from the holding potential) of total I_Ca_ recorded from the holding potential of −90 mV (not adjusted for junctional potential which was about −10 mV) (a), of I_Ca-L_ recorded from the holding potential of −50 mV (b), of currents recorded from the holding potential of −50 mV after the application of 100uM Cd^2+^ (c), of differential currents between total I_Ca_ and I_Ca-L_ (d), and of Cd^2+^-sensitive currents (difference current obtained by subtracting total I_Ca_ from Cd^2+^ insensitive current, presumably I_Ca-L_, (e). In B, please note that the fast downward currents recorded were capacitance transients (currents charging and discharging the membrane) when we hyperpolarized the cell from −50 mV to −90 mV~-60 mV. (**f)**. Current (i)-Voltage (v) relationships of total I_Ca_, I_Ca_ recorded with the holding potential of −50 mV, differential currents between total I_Ca_ and I_Ca-L_, Cd^2+^-insensitive and Cd^2+^ sensitive currents. The I–V curves of total I_Ca_, I_Ca_ recorded with the V_h_ of −50 mV and Cd^2+^ sensitive currents were overlapping.

In contrast, in a failing HLVM without I_Ca-LVA_ as shown in Figure 2, 100 µM Cd^2+^ completely abolished all Ca^2+^ currents recorded with a Vh of −90 mV (**Figure 2**a,c). There was no difference between total I_Ca_ measured with the V_h_ of −90 mV (Figure 2a), the L-type Ca^2+^ current measured with the V_h_ of −50 mV (Figure 2b) and the Cd^2+^-sensitive currents (Figure 2e). The I–V relationships of total I_Ca_, I_Ca-L_ and the Cd^2+^ sensitive currents were overlapping very well (figure 2f). The threshold for activation was −20 mV and the density was greater than that of the failing myocyte in Figure 1, suggesting this I_Ca-L_ was more likely mediated by Cav1.2 only while I_Ca-L_ in Figure 1 could contain both Cav1.2 and Cav1.3 currents that were down-regulated by heart failure.

### I_Ca-LVA_ current in 2 failing HLVMs is not sensitive to TTX and inactivates faster than I_Ca-L_

**Figure 3.**
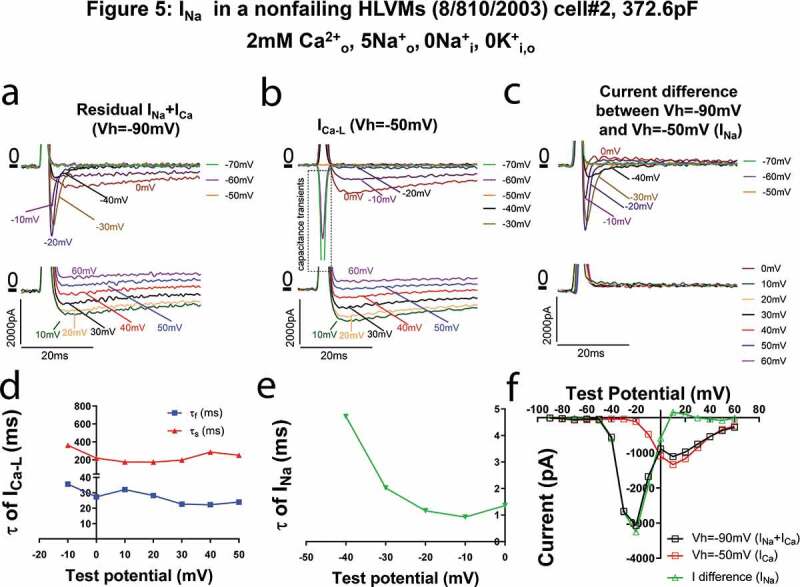
I_Ca-LVA_ in a HLVM of a failing human heart due to primary pulmonary hypertension is insensitive to 100 µM TTX and inactivates faster than I_Ca-L_. The HLVM was incubated in Ca^2+^ current recording perfusate containing 100 µM TTX. **A-C**. Current traces at different test voltages (in a 10 mV-increment from the holding potential) of total I_Ca_ recorded from the holding potential of −90 mV (a), of I_Ca-L_ from the holding potential of −50 mV (b), of differential currents between currents recorded with the holding potential of −90 mV and currents recorded with the holding potential of −50 mV (c). In B, please note that the fast downward currents recorded were capacitance transients (currents charging and discharging the membrane) when we hyperpolarized the cell from −50 mV to −90 mV~-60 mV. (d). Fast and slow time constants (τ_f_ and τ_s_) of I_Ca-L_ at different test potentials. (e). Time constants (τ) of I_Ca-LVA_ at different test potentials. (f). The current (i)-Voltage (v) relationships of total I_Ca_, I_Ca_ recorded with the holding potential of −50 mV (I_Ca-L_), differential currents between currents recorded with the holding potential of −90 mV and currents recorded with the holding potential of −50 mV (I_Ca-LVA_).

We found another cell with I_Ca-LVA_ like current which appeared at the test potential of −40 mV from the holding potential of −90 mV in the presence of tetrodotoxin (TTX, 100 µM). Total I_Ca_, I_Ca-L_ (V_h_ = −50 mV) and I_Ca-LVA_ (I_Ca_ difference between total I_Ca_ and I_Ca-L_) recorded in this TTX containing solution were shown in **Figure 3**a–c. While I_Ca-L_ recorded at Vh = −50 mV could be better fit with a double exponential decay equation, I_Ca-LVA_ was well fit with a single exponential decay equation. The slow and fast time constants (τ_s_ and τ_f_) of I_Ca-L_ and time constant (τ) of I_Ca-LVA_ were shown Figure 3d and e, respectively. The time constants (τ) for I_Ca-LVA_ were smaller than the slow time constants (τ_s_) of I_Ca-L_ and similar to fast τ values of I_Ca-L_, consistent with a previous report [33].

**Figure 4.**
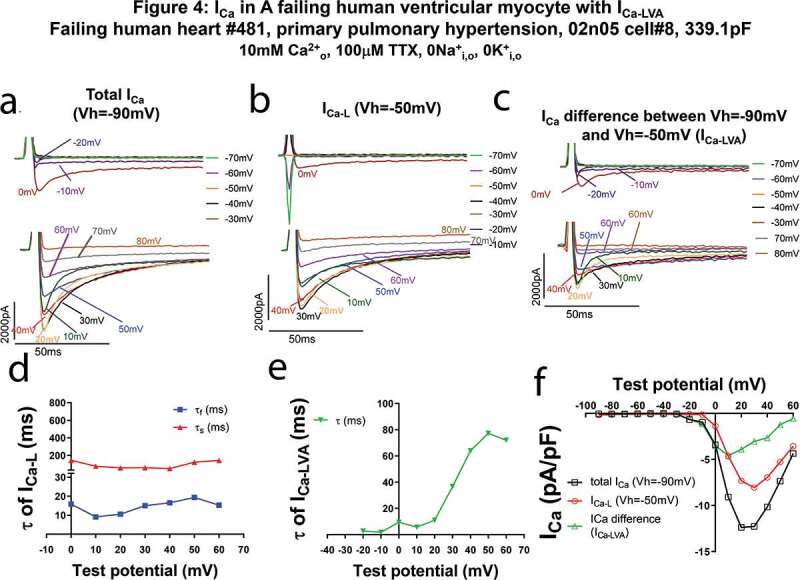
I_Ca-LVA_ in a HLVM of a failing human heart due to primary pulmonary hypertension recorded with 10 mM extracellular Ca^2+^ is insensitive to 100 µM TTX. A FHLVM was incubated in Ca^2+^ current recording perfusate containing 10 mM Ca^2+^ and 100 µM TTX. (a–c). Current traces at different test voltages (in 10 mV increment from the holding potential) of total I_Ca_ recorded from the holding potential of −90 mV (a), of I_Ca_ recorded with the holding potential of −50 mV (b, presumably I_Ca-L_), and of differential currents between total I_Ca_ and I_Ca-L_ (c). In B, please note that the fast downward currents recorded were capacitance transients (currents charging and discharging the membrane) when we hyperpolarized the cell from −50 mV to −90 mV~-60 mV. (d). Fast and slow time constants (τ_f_ and τ_s_) of I_Ca-L_ at different test potentials. (e). Time constants (τ) of I_Ca-LVA_ at different test potentials. (f). The current (i)-Voltage (v) relationships of total I_Ca_, I_Ca_ recorded with the holding potential of −50 mV (I_Ca-L_), and differential currents between currents recorded with the holding potential of −90 mV and currents recorded with the holding potential of −50 mV (I_Ca-LVA_). The shift of I–V curves to more positive directions was due to high extracellular Ca^2+^ causing the surface charge screening effect.

**Figure 6.**
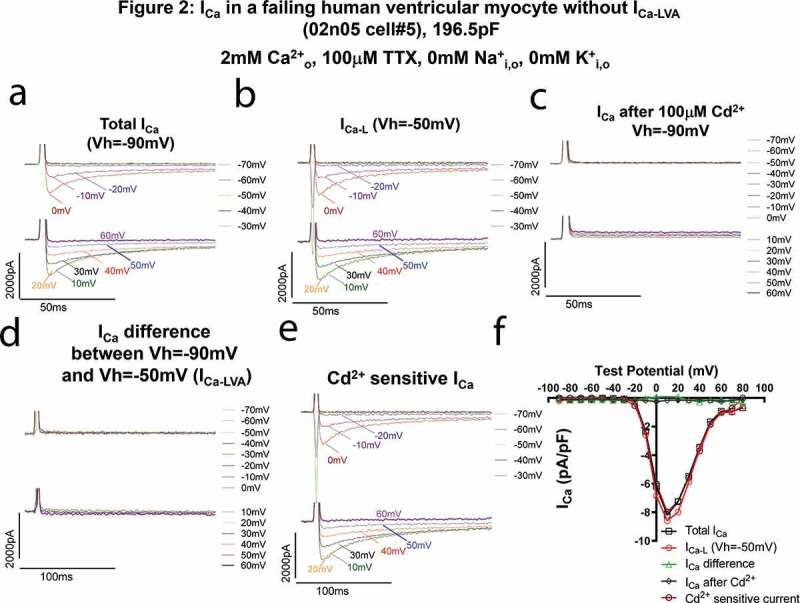
I_Ca-T_ mediated by human T-type Ca^2+^ channel Cav3.2/α1 H in transgenic mouse ventricular myocytes cardiac-specifically overexpressing α1 H. (a–c). Current traces at different test voltages (in 10 mV increment from the holding potential) of total I_Ca_ recorded from the holding potential of −90 mV (a), of I_Ca_ recorded with the holding potential of −50 mV when I_Ca-T_ was inactivated (b, presumably I_Ca-L_), of differential currents between currents recorded with the holding potential of −90 mV and currents recorded with the holding potential of −50 mV (presumably I_Ca-T_, (c). In B, please note that the fast downward currents recorded were capacitance transients (currents charging and discharging the membrane) when we hyperpolarized the cell from −50 mV to −90 mV~-60 mV. (d). Fast and slow time constants (τ_f_ and τ_s_) of I_Ca-L_ at different test potentials. (e). Time constants (τ) of I_Ca-T_ at different test potentials. I_Ca-T_ could be fit well with single exponential decay. (f). The current (i)-voltage (v) relationships of total currents containing both I_Ca_ and I_Na_, I_Ca_ recorded with the holding potential of −50 mV (I_Ca-L_), differential currents between currents recorded with the holding potential of −90 mV and currents recorded with the holding potential of −50 mV (presumably I_Na_).

We also tried to record I_Ca-LVA_ current with a high extracellular Ca^2+^ (10 mM) bath solution containing 0 Na^+^ and 0 K^+^, and 100 µM TTX in FHVMs and found one FHVM with I_Ca-LVA_. The high extracellular Ca^2+^ shifted the activation of the L-type and I_Ca-LVA_ currents to more positive voltages due to the surface charge screening effect [34] (Figure 4). The decay of I_Ca-L_ and I_Ca-LVA_ was fit with a double- or single- exponential decay function, as shown in Figure 4d and e. It seemed that the τ values here were smaller than those in Figures 1 and 6, probably due to high extracellular Ca^2+^ (10 mM). The peak I_Ca-LVA_ was −4.58 pA/pF (Figure 4f), greater than the amplitudes of I_Ca-LVA_ in the other two FHVMs recorded with bath solution containing 2 mM Ca^2+^.

### I_Ca-LVA_ current in failing HVMs inactivates slower than I_Na_

**Figure 5.**
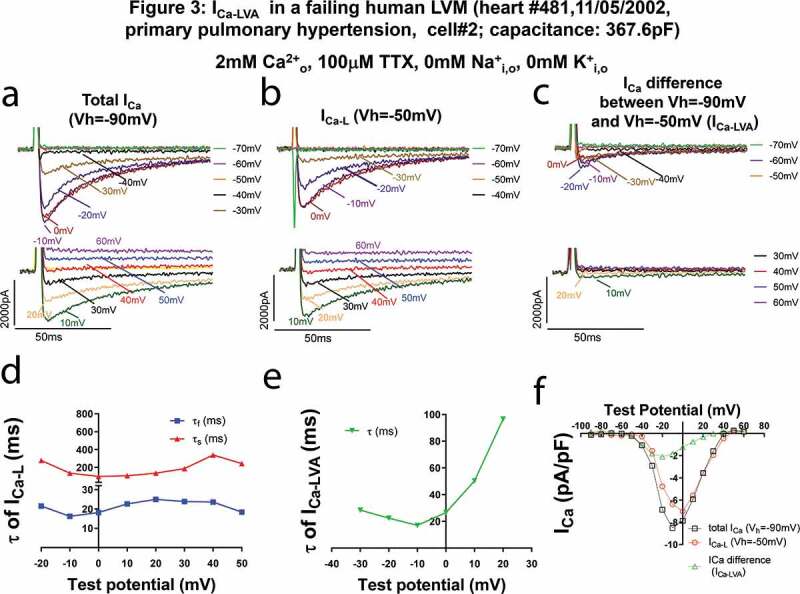
Sodium current (I_Na_) in a nonfailing HLVMs recorded with 5 mM extracellular Na^+^ added to Ca^2+^ current recording solution. (a–c). Current traces recorded at different test voltages (in 10 mV increment from the holding potential) of total I_Ca_ with I_Na_ current contamination recorded with the holding potential of −90 mV (a), I_Ca_ recorded with the holding potential of −50 mV when sodium channels were inactivated (b, presumably I_Ca-L_), differential currents between currents recorded with the holding potential of −90 mV and currents recorded with the holding potential of −50 mV (presumably I_Na_, (c). In B, please note that the fast downward currents recorded were capacitance transients (currents charging and discharging the membrane) when we hyperpolarized the cell from −50 mV to −90 mV~-60 mV. (d). Fast and slow time constants (τ_f_ and τ_s_) of I_Ca-L_ at different test potentials. (e). Time constants (τ) of I_Na_ at different test potentials. I_Na_ could be fit well with a single exponential decay function. τ values were much smaller than τ_f_ of I_Ca-L_ or I_Ca-T_. (f). Current (i)-Voltage (v) relationships of total currents containing both I_Ca_ and I_Na_, I_Ca_ recorded with the holding potential of −50 mV (I_Ca-L_), and differential currents between currents recorded with the holding potential of −90 mV and currents recorded with the holding potential of −50 mV (presumably I_Na_).

We further compared the properties of I_Ca-LVA_ current in the human ventricular myocytes with I_Na_ in a nonfailing HLVMs recorded with low concentration of Na^+^ (Figure 5). In current traces recorded at Vh = −90 mV (I_Ca_+I_Na_), there were large and quickly inactivated currents (Figure 5a), which was eliminated by the V_h_ = −50 mV (Figure 5b, note that the downward currents in Figure 5b were the capacitance transient currents when hyperpolarizing the cell from −50 mV. They were neither I_Ca-LVA_ nor I_Na_). I_Na_ was obtained by subtracting I_Ca_ from total current and its inactivation was very fast (Figure 5c and f). I_Na_ completely returned to baseline within 10 ms and the time constants were <5 ms at all test potentials (Figure 5c and e), which were an order smaller than the time constants of I_Ca-LVA_ (Figure 5e versus Figure 3e) and the fast time constants of I_Ca-L_ (Figure 5d). These results suggest that the I_Ca-LVA_ currents observed in failing human ventricular myocytes are not I_Na_.

### I_Ca-LVA_ current in comparison with I_Ca-T_ mediated by Cav3.2/α1 H in mouse ventricular myocytes

We intended to compare properties of I_Ca-LVA_ recorded in HLVMs and those of I_Ca-T_ in transgenic mouse ventricular myocytes expressing human Cav3.2/α1 H. In VMs isolated from these transgenic mice, I_Ca-T_ can be separated by holding the cell at −90 mV versus at −50 mV (Figure 6a–c, f). The inactivation time constants (Figure 6e) and voltage dependency (figure 6f) of I_Ca-T_ mediated by human Cav3.2/α1 H in mouse VMs were like I_Ca-LVA_ current observed in human VMs. However, there was an extra sustained component in I_Ca-LVA_ in HLVMs in Figures 1, 3 and 4, which could be mediated by Cav1.3.

## Discussion

### Few human LVMs have I_Ca-LVA_ current

The existence of I_Ca-T_ current in human ventricular myocytes were explored in myocytes isolated from failing or nonfailing human hearts and no I_Ca-T_ was found previously [14]. No Cav1.3 I_Ca-LVA_ was found in human ventricular myocytes previously [35] but Cav1.3 protein was shown in human ventricular tissue [23]. In this study, we examined substantial number of human left ventricular myocytes from nonfailing hearts, failing hearts and failing hearts with LVAD support to find I_Ca-LVA_ with whole-cell voltage-clamp technique. We found I_Ca-LVA_ insensitive to 100 µM TTX, having the activation threshold at ~-50 mV, peaked around −30 mV and inactivating at rates faster than Cav1.2 I_Ca-L_ but slower than I_Na_, in 3 LVMs from 2 failing human hearts but not from nonfailing and failing with LVAD-support human hearts. These properties were different from Cav1.2 L-type Ca^2^^+^ currents or Na^+^ currents and more consistent properties of I_Ca-LVA_ current (Cav1.3 or Cav3 or both). However, the existence of I_Ca-LVA_ was found in only few HLVMs of few human hearts. I_Ca-LVA_ in 1 HLVM was detected in the presence of 100 µM CdCl_2_, suggesting that in that cell I_Ca-LVA_ was more likely I_Ca-T_, as Cav1.3 mediated I_Ca_ can be well blocked by 100 µM CdCl_2_ [25]. However, in the other two cells with I_Ca-LVA_, we did not have the chance to determine I_Ca-LVA_ sensitivity to Cd^2^^+^ or Ni^2^^+^. Thus we could not determine which subtype of LVA Ca^2+^ channels (Cav1.3 or Cav3.1 or Cav3.2 or Cav3.3) [36] was expressed in these myocytes.

### What kind of human LVMs were expressing I_Ca-LVA_?

Firstly, it is clear that I_Ca-LVA_ was only found in nonischemic failing HLVMs. However, statistical analysis did not identify a significant correlation between the expression of I_Ca-LVA_ and the disease states (F, NF or F-LVAD) or between I_Ca-LVA_ and etiologies (ischemic or nonischemic). Our data suggest that the existence of I_Ca-LVA_ in adult human LVMs is rare and happened to be in nonischemic failing human LVMs.

Secondly, these HLVMs with I_Ca-LVA_ were probably not myocytes with automaticity. Within the ventricles, I_Ca-LVA_ current could be present in Purkinje cells. However, according to our isolation procedure, human LVMs used in this study were from the middle wall where Purkinje cells were absent [37]. In addition, these myocytes might not be Purkinje cells because they had a rod shape, dense myofibrils and clear striations while human Purkinje cells are long, narrow spindle-shaped and with sparse myofibrils [38]. However, we could not rule out that these myocytes could be transition myocytes between Purkinje myocytes and ventricular myocytes.

### What are the roles of I_Ca-LVA_ in failing human LVMs?

In fetal hearts, TTCCs clearly participate in cardiac excitation-contraction coupling [6]. We showed that in adult mouse myocytes, TTCCs do not play a significant role in inducing excitation-contraction coupling process. Nor does Ca^2+^ influx through TTCCs effectively load the sarcoplasmic reticulum [16]. In normal adult ventricular myocytes, TTCC expression is mostly absent except for few species [12,13].

TTCC expression reappears in stressed myocardium [2,19]. T-type Ca^2+^ current has been reported to be increased in genetically hypertrophied hamster hearts [39]. Multiple hormones (e.g., angiotensin II, aldosterone), growth factors (e.g., IGF-1) and transcription factors (e.g., NRSF and Nkx2.5) involved in cardiac disease progression have been shown to regulate the expression of TTCCs in cardiomyocytes [2]. However, the pathological significance of TTCC reappearance in diseased hearts is not clear. It may modulate the excitation-contraction process by enhancing the amplification of Ca^2+^-induced Ca^2+^ release and modestly increasing SR Ca^2+^ content [15]. The loss of Cav3.1 in mouse hearts exacerbated cardiac function after myocardial infarction [40]. Cav3.2 is necessary for cardiac hypertrophy [2] but the increase of Ca^2+^ influx through Cav3.1 antagonizes cardiomyocyte hypertrophy [21] rather than promotes cardiomyocyte hypertrophy as increased Ca^2+^ influx through the LTCC does [41]. Additionally, TTCC reappearance may contribute to cardiac arrhythmias observed in stressed hearts because the T- type Ca^2+^ channel blockers reduced arrhythmias independent of their L-type Ca^2+^channel blocker activity [42]. Another possibility is that TTCC re-expression is simply part of re-expression of the fetal gene program in diseased hearts. In addition, the expression of TTCCs is associated with cell cycle [2] and we could not rule out these human LVMs with I_Ca-LVA_ were also associated with cell cycle activity.

If the I_Ca-LVA_ observed in our study was Cav1.3 mediated, its significance needs to be further determined. It has been reported that in atrial myocytes Cav1.3 couples with small conductance Ca^2+^-activated K^+^ channels and the loss of Cav1.3 prolongs the action potential [23]. However, the role of Cav1.3 in the ventricles has not been well studied and the physiological and pathological relevance of Cav1.3 in HLVMs warrants further study.

Though we only found 1 out of 7 LVMs from a failing heart with idiopathic cardiomyopathy and another 2 out of 8 LVMs from another failing heart with primary pulmonary hypertension had I_Ca-LVA_, the absolute number of LVMs expressing I_Ca-LVA_ in these two specific hearts could be large as the total myocyte number in human hearts is huge. It is highly likely that TTCCs in these two hearts play some physiological and/or pathological roles.

### Limitations

Our study indicates that a very minimal but still detectable amounts of human myocytes had I_Ca-LVA_. While all 3 LVMs with I_Ca-LVA_ were from failing human hearts, statistically we did not detect difference in possibilities of I_Ca-LVA_ expression among nonfailing, failing and failing with LVAD support hearts. On the other hand, as the detection method used was low throughput and human left ventricle has a huge number of cardiomyocytes, the hearts deemed without I_Ca-LVA_ in LVMs may actually contain few myocytes with I_Ca-LVA,_ which could be easily missed by our low throughput detection. It is also possible that the expression/re-expression of I_Ca-LVA_ in VMs were restricted in a specific area of the ventricles and the midwall of the free wall of the LV surveyed by us had less percentage of myocytes expressing I_Ca-LVA_. Furthermore, there was rundown of I_Ca-T_ [32] and the amplitude of I_Ca-LVA_ could be too small to be confidently measured in some LVMs. Taken together, this study may underestimate the percentage of hearts with I_Ca-LVA_ and the re-expression of I_Ca-LVA_ in some failing human hearts may play important pathological roles. At last, this study was to retrospectively examine the existence of I_Ca-LVA_ and key experiments for determining the nature of the current, such as the application of low concentrations of Ni^2+^, Cd^2+^ and dihydropyridine blockers, were not performed. The biophysical properties, including voltage dependent activation and inactivation, and time dependent decay, of Cav1.3 and Cav3 Ca^2+^ channels overlap [2,22]. Therefore, we could not determine if these currents were mediated by Cav1.3 or Cav3 or both.

### Conclusion

We recorded a low voltage-activated Ca^2+^ currents in few human ventricular myocytes of few human hearts that happened to be nonischemic failing.
